# Chronic caffeine consumption on clinical TMS outcomes: A naturalistic retrospective analysis

**DOI:** 10.1016/j.transm.2025.100199

**Published:** 2025-11-07

**Authors:** Jamie Kweon, Prem Ganesh, Joshua C. Brown

**Affiliations:** a Brain Stimulation Mechanisms Laboratory, Division of Depression and Anxiety Disorders, McLean Hospital, Belmont, MA 02478, United States; b Department of Psychiatry, Harvard Medical School, Boston, MA 02115, United States

**Keywords:** TMS, Caffeine, Naturalistic, Depression

## Abstract

Health and behavioral factors likely influence repetitive transcranial magnetic stimulation (rTMS) effectiveness, though clear support is lacking. Since habitual caffeine use has been found to impair corticomotor plasticity, we hypothesized that clinical benefits may likewise be impaired. We performed a retrospective analysis (n = 179) from the McLean Hospital TMS Clinic to explore the effect of self-reported caffeine use on TMS clinical outcomes. Self-report depression and anxiety scales from caffeine users and non-users were compared with Wilcoxon-rank-sum test and response and remission rates with chi-square; Spearman’s was used to correlate correlation caffeine quantity with degree of clinical change. Both groups experienced a significant reduction in depressive symptoms as expected. Contrary to our hypothesis, however, we found no differences between caffeine users and non-users in total symptom reduction or response and remission rates, nor did we find a relationship between caffeine quantity and clinical change. This naturalistic analysis suggests that chronic caffeine consumption does not influence treatment outcomes in clinical rTMS for depression.

## Introduction

Factors that guide patient selection or impact effectiveness of repetitive transcranial magnetic stimulation (rTMS) could have an immediate effect on clinical care. Health factors like sleep (Kweon et al., 2024), exercise (Schuch et al., 2018), or engagement in therapy (Donse et al., 2018) have known benefits while some substances have known detriments (DePamphilis et al., 2024; Turco et al., 2020). Medications have varied reported effects, including beneficial effects of stimulants (Wilke et al., 2022), impairing effects from antipsychotics (Hebel et al., 2020) and mixed reports on benzodiazepine use (Deppe et al., 2021; Fitzgerald et al., 2020; Hunter et al., 2019; Kaster et al., 2019). Most common among all substances is caffeine, consumed by 85 % of the U.S. population (Meredith et al., 2013), yet its effects are arguably the least studied.

Caffeine is a competitive antagonist at adenosine receptors, blocking inhibition and, in turn, resulting in increased cortical excitability and alertness (van Dam et al., 2020). Its ability to influence cortical excitability as a relatively weaker stimulant has made it particularly relevant as a modulator in brain stimulation; for example, caffeine is used to lower seizure threshold in electroconvulsive therapy (Nyhuis et al., 2024). The potential impact of caffeine on rTMS response is highlighted by a recent study reporting blunted corticomotor plasticity in caffeine users after single sessions with d-cycloserine or placebo (Vigne et al., 2023). Similarly, acute caffeine administration impaired transcranial alternating current stimulation (tACS)-induced plasticity (Zulkifly et al., 2020) and quadripulse stimulation (QPS) (Hanajima et al., 2019). However, a sham-controlled clinical trial delivering sessions twice daily for 20–30 total sessions to the dorsomedial prefrontal cortex (dmPFC) saw that habitual caffeine use was positively correlated with degree of symptom improvement (Frick et al., 2021). This discrepancy is reflective of the caffeine literature which has produced mixed results. In animals, as another example, caffeine has both disrupted plasticity (Blaise et al., 2018) and rescued deficits (Alhaider et al., 2010).

Given the lack of consensus across more basic studies and absence of naturalistic reports on the subject, we sought to understand the effect of caffeine on naturalistic TMS. To address this gap in knowledge, we present a naturalistic study examining the potential relationship between chronic caffeine consumption and treatment outcomes for TRD. We hypothesized that through impaired plasticity, caffeine users will have reduced clinical improvement compared to non-caffeine users following naturalistic course of TMS.

## Methods

We retrospectively analyzed 266 records from May 2023 to March 2025 collected through the McLean Hospital outpatient TMS clinic and covered under the McLean Hospital Institutional Review Board. Only patients receiving their first course of TMS were included. Patients that received TMS for any disorder other than MDD, had a baseline clinical score < 5 on either the Quick Inventory of Depressive Symptomology Self-Report (QIDS-SR16) or Patient Health Questionnaire 9-Item (PHQ-9), or received less than 20 treatments were excluded, leaving 179 patients.

### Questionnaires

The McLean Hospital TMS clinic collected the QIDS-SR16, PHQ-9, and Generalized Anxiety Disorder 7-Item (GAD-7). Patients completed clinical scales before their course and after every five to ten treatments. Clinical response was defined as a 50 % or greater score reduction. Remission was defined as a final score of ≤ 5 on QIDS-SR16 or ≤ 4 on PHQ-9. Patients also completed a caffeine questionnaire during their initial consultation visit, which asks i) whether they used caffeine products, ii) the type of products, iii) frequency of use, and iv) servings per use. All individuals had a diagnosis of treatment-resistant depression with at least two antidepressant trials.

### TMS protocol

Of the 179 patients included in this analysis, most patients (134, 74.9 %) were treated on a BrainsWay device with H1 coil, which consisted of 18-Hz stimulation for 1980 pulses five days per week for up to 36 consecutive treatments. About 25.1 % (45) of patients were treated on a MagVenture device with B70 figure-8 coil, with either left-hemisphere intermittent theta-burst stimulation (iTBS) (30, 16.8 %), right-hemisphere 1-Hz or continuous (c)TBS (12, 6.7 %), or bilateral (3, 1.7 %). In cases of poor tolerability to BrainsWay (14, 7.8 %), patients were switched to the MagVenture device. There were no statistical differences between TMS protocols in response (QIDS-SR16: p = 0.299; PHQ-9: p = 0.507; GAD-7: p = 0.133) nor remission rates (QIDS-SR16: p = 0.550; PHQ-9: p = 0.721; GAD-7: p = 1.00) across all outcome measures.

### Statistical analysis

To test whether there were differences in TMS outcomes by caffeine users, continuous variables (baseline and final QIDS-SR16, PHQ-9, and GAD-7 scores) were first determined to have nonnormal distribution by Shapiro Wilk test. Score comparisons at timepoints or between caffeine users were therefore analyzed using Wilcoxon rank-sum test. To directly compare treatment response between groups, Kruskal-Wallis tests were performed on improvement scores (baseline minus final scores) to assess whether caffeine users and non-users had different distributions of treatment response. Chi-square tests were used to identify potential difference in rates of responders or remitters by caffeine group. Tests were performed for each questionnaire.

We conducted a sensitivity analysis to examine whether TMS device type or intolerance patterns affected the relationship between caffeine use and treatment outcomes. Patients were categorized based on their TMS treatment history: BrainsWay-only (received only BrainsWay treatment), MagVenture-only (received only MagVenture treatment), or device switchers (started on BrainsWay but switched to MagVenture due to intolerance). We first tested whether overall treatment outcomes differed by starting device using Kruskal-Wallis tests. Next, we examined caffeine effects separately within each device group to determine if caffeine associations were device-specific. We then performed an analysis excluding device switchers to test whether intolerance-related switching influenced our findings. Additionally, we compared treatment outcomes between device switchers and non-switchers. Finally, we tested for caffeine × device interactions by creating four groups (caffeine users on BrainsWay, non-users on BrainsWay, caffeine users on MagVenture, non-users on MagVenture) and comparing treatment improvements across all groups using Kruskal-Wallis tests.

Caffeine products were reported: coffee, tea, energy drinks, soda, caffeine pills, Excedrin, pre-workout supplements, and dark chocolate (Table 1). Consumption frequency and quantity were assessed by two questions on the caffeine questionnaire: “How many days per week do you take caffeine?” and “How many servings per day (on average) do you take caffeine? To calculate a total servings per week, the response values to the two questions were multiplied to create a new continuous variable. We tested whether there was a relationship between servings per week and percent change in clinical scales using Spearman’s rank correlation test. Statistical significance for analyses was defined at p < 0.05.

## Results

**Fig. 1.**
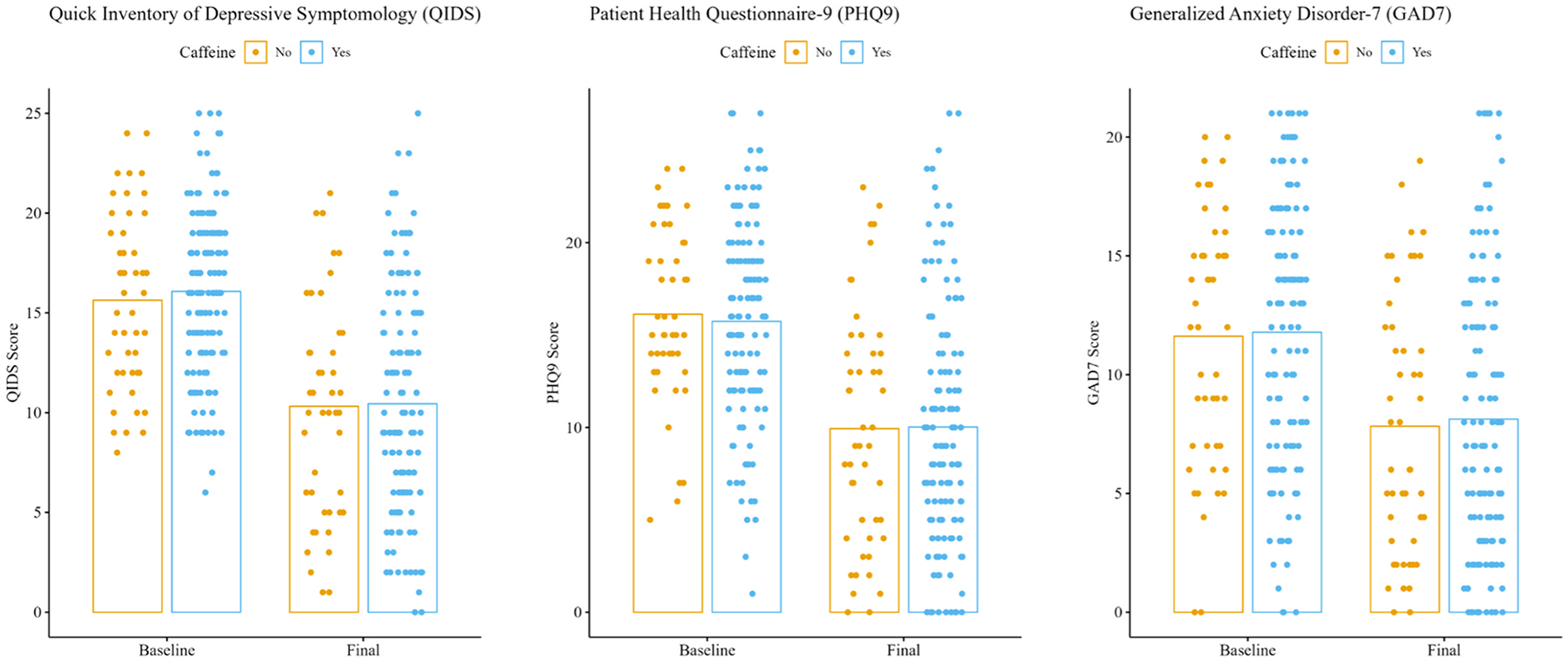
Clinical scales before and after TMS course by caffeine user status. No differences were detected between caffeine groups at any timepoint across all scales.

Analysis consisted of 179 patients (62.6 % F, mean age = 44.9) with 47 non-caffeine users and 132 caffeine users. 51 % of non-caffeine users were inpatient at consult and 49 % were inpatient during treatment compared to only 28 % and 19 % respectively for caffeine users (see supplemental Table 1). Across all patients, there was a significant improvement from baseline to final TMS treatment in QIDS-SR16 (Z = −9.16, p < .001), PHQ-9 (Z = −8.55, p < .001), and GAD-7 (Z = −5.99, p < .001) scores. There was no difference (Fig. 1) at baseline between caffeine groups for all three scales (QIDS-SR16: W = −.623, p = .533; PHQ-9: W= −.395, p = .693; GAD-7: W = −.121, p = .904) nor at final treatment (QIDS-SR16: W = −.020, p = .984; PHQ-9: W = −.079, p = .937; GAD-7: W = −.207, p = .836). Kruskal-Wallis tests comparing the distributions of improvement scores between caffeine users and non-users revealed no significant differences across all outcome measures (QIDS-SR16: χ^2^ = 0.005, p = .942; PHQ-9: χ^2^ = 0.294, p = .588; GAD-7: χ^2^ = 0.201, p = .654). Caffeine users and non-users did not significantly differ in percent improvement after rTMS on the QIDS-SR16 (p = .816) nor the PHQ-9 (p = .282).

Among 179 patients, 120 (67 %) received BrainsWay-only treatment, 43 (24 %) received MagVenture-only treatment, and 14 (7.8 %) switched from BrainsWay to MagVenture due to intolerance. Device type did not significantly affect treatment outcomes overall (QIDS-SR16 p = 0.78, PHQ-9 p = 0.84, GAD-7 p = 0.08). Critically, caffeine effects remained non-significant when analyzed separately within each device group: among BrainsWay-only patients (QIDS-SR16: p = 0.88, PHQ-9: p = 0.69, GAD-7: p = 0.38) and MagVenture-only patients (QIDS-SR16: p = 0.28, PHQ-9: p = 0.50, GAD-7: p = 0.64). When excluding the 14 device switchers entirely, caffeine effects remained non-significant (QIDS-SR16: p = 0.54, PHQ-9: p = 0.44, GAD-7: p = 0.32). No significant caffeine × device interactions were observed (QIDS-SR16: p = 0.64, PHQ-9: p = 0.88, GAD-7: p = 0.34).

Table 2 provides summary of patient characteristics. Total QIDS-SR16 response rate was 36.9 % and total remission rate was 23.2 %. Within the caffeine users, we found a 37.1 % response and 21.2 % remission rate, while within non-caffeine users we found a 36.2 % response and 25.5 % remission rate. Chi-square analysis revealed no difference between the two groups’ rates of response (χ^2^ =.001, p = .968) nor remission (χ^2^=.517, p = .472). Total PHQ-9 response rate was 41.3 % with a remission rate of 27.9 %. Caffeine users had PHQ-9 response rates of 41.7 % and remission rates of 26.5 %, while non-caffeine users had response rates of 40.4 % and remission rates of 31.9 %. Chi-square using PHQ-9 calculated outcomes demonstrated consistent results (response: χ^2^=.008, p = .93; remission: χ^2^=.753, p = .385).

Participants consumed caffeine on average 5.85 days per week with a mean 1.8 servings per day. Average total servings per week was 11.0 ± 7.11. We found no correlation between servings per week and QIDS-SR16 % change (r_s_=.075, p = .225) nor PHQ-9 % change (r_s_ =.053, p = .39) using Spearman’s Correlation.

## Discussion

In this first naturalistic report of chronic caffeine effects on clinical TMS effectiveness, we found that contrary to our hypothesis, both caffeine users and non-users had nearly identical responses; nor was there a relationship between quantity and frequency of caffeine consumption with clinical change. While caffeine has been shown to impair brain stimulation-mediated plasticity with acute (Hanajima et al., 2019; Zulkifly et al., 2020) and chronic consumption (Vigne et al., 2023), this does not appear to extend to clinical benefit. There are several possibilities for this. First, each of these studies used a different protocol from our naturalistic cohort, which may interact differently with caffeine’s effects on plasticity. Second, plasticity in the motor cortex, where each of these studies was conducted, may also differ from the dlPFC in plasticity. Moreover, while the more focused examination of corticomotor plasticity may respond to classical Hebbian plasticity, the more distributed prefrontal cortex networks could involve more metaplastic homeostatic ‘buffering’ effects (Li et al., 2019). Third, the assumption that plasticity underlies clinical effects may be incorrect (Brown et al., 2020, 2021). Finally, clinical presentations are multifactorial and arguably more susceptible to extraneous influences in contrast with relatively simple plasticity measures.

We note that our overall response rates (37 % QIDS-SR16, 37 % PHQ-9) and remission rates (26 % QIDS-SR16, 28 % PHQ-9) are lower than some naturalistic reports. We cannot determine with certainty the reason for this, but 26.1 % of our patients were inpatient during treatment (18.9 % of caffeine users and 48.9 % of non-caffeine users by group), see supplemental table 1) and 25.1 % are prior ECT or ketamine non-responders (22 % and 34 % by caffeine use), suggesting a fairly treatment resistant population. Given these differences between caffeine use among inpatients and outpatients, and the possibility that inpatient status affected access to regular caffeine use, we separately analyzed the effect of caffeine status on clinical outcomes by comparing inpatients to inpatients and outpatients to outpatients from age/sex-matched subgroups. We found no differences among these subgroups (inpatients: p = .65 for QIDS-SR16 and p = .35 for PHQ-9 and outpatients: p = .86 for QIDS-SR16 and p = .63 for PHQ-9, see supplemental table 1). Ultimately, these data are consistent with our stated conclusions that caffeine use did not alter clinical response.

Several **limitations** must be considered against our null findings. First, inherent to naturalistic studies, we did not randomize or control for confounds. We therefore cannot account for the influence of medications; future studies would benefit from analyses on patients also taking psychostimulants. Another challenge is that caffeine levels were merely gross estimates: We do not know the biological concentrations of caffeine, nor did we supervise administration to ensure consistent dosages. An interesting future direction would be the study of differential optimal dosages by different rTMS parameters, which can influence the direction and strength of plasticity induction (Caulfield & Brown, 2022). Along these lines, caffeine concentrations differ across various products and caffeine metabolism is variable with half-lives ranging from 1.5 to 9.5 h (Brachtel & Richter, 1992). The assessment also did not capture timing of caffeine consumption, so we cannot separate the effects of chronic and acute use. Further, for those who reported using more than one caffeine product, the questionnaire did not capture the specific frequency and quantity of consumption for each individual product. The strength of these approaches is that our approach is in line with real world practice, and therefore, if there were an effect of caffeine, that effect might be diluted out in the context of typical use habits and varied metabolism. It is also unlikely that caffeine levels were a major confound because no amount of caffeine appeared to have an effect. Nevertheless, to understand the effect of caffeine on TMS, a randomized controlled trial with serum concentrations would be necessary.

We conclude from these data that it is not necessary to restrict caffeine use in patients who are embarking on a course of TMS as it appears unlikely to have an effect.

## Supplementary Material

1

## Figures and Tables

**Table 1:** Summary of reported caffeine products. Majority of caffeine users reported more than one product. General serving sizes and caffeine content were cited from the following sources: (Chin et al., 2008; Gilbert et al. 1976; Jagim et al., 2022; Rubio et al., 2022; van Dam et al., 2020).

Product Type	Total Number of Users	General Serving Size	General Caffeine content per serving	Description
Coffee	204	8 fl oz	90–200 mg	
Tea	122	8 fl oz	14–61 mg	
Energy Drinks	40	8.4 fl oz	50–160 mg	
Soda	84	12 fl oz	20–55 mg	
Caffeine Pills	2	1 tablet	200 mg	
Other	4	NA	NA	Dark chocolate, Excedrin, protein power

**Table 2:** Patient characteristics.

Demographics	Caffeine Non-Users (n = 47)	Caffeine Users (132)	p
Age (mean ± SD)	45.0 ± 21.1	44.9 ± 17.3	.982
Sex (n, % Female)	28 (59.6 %)	84 (63.6 %)	.712
**Clinical Severity (mean ± SD)**			
Baseline QIDS-SR16	16.1 ± 4.12	15.6 ± 4.38	.552
Final QIDS-SR16	10.4 ± 5.64	10.3 ± 5.35	.890
Raw Change QIDS-SR16	−5.6 ± 5.7	−5.3 ± 4.3	.942
Percent Change QIDS-SR16	33.4 ± 35.0	34.6 ± 31.2	.816
Baseline PHQ-9	15.7 ± 5.50	16.1 ± 4.75	.648
Final PHQ-9	10.0 ± 6.47	9.94 ± 6.37	.937
Raw Change PHQ-9	−5.7 ± 6.4	−6.2 ± 5.5	.588
Percent Change PHQ-9	29.9 ± 59.7	38.1 ± 38.3	.282
Baseline GAD-7	11.8 ± 5.58	11.6 ± 5.37	.855
Final GAD-7	8.13 ± 5.76	7.83 ± 5.46	.751
Raw Change GAD-7	−3.6 ± 5.0	−3.8 ± 4.6	.654
Percent Change GAD-7	27.4 ± 47.9	35.1 ± 41.7	.310
**Treatment Outcomes (n, %)**			
QIDS-SR16 Response	49, 37.1 %	17, 36.2 %	.968
QIDS-SR16 Remission	28, 21.2 %	19, 25.5 %	.472
PHQ-9 Response	55, 41.7 %	12, 40.4 %	.930
PHQ-9 Remission	35, 26.5 %	15, 31.9 %	.385

## Appendix A. Supporting information

Supplementary data associated with this article can be found in the online version at doi:10.1016/j.transm.2025.100199.
