# Supplementary material for: Chronic caffeine consumption on clinical TMS outcomes: A naturalistic retrospective analysis
Source: Transcranial Magn Stimul. Author manuscript; Available in PMC 2025 Dec 4. (PMC12674203; doi:10.1016/j.transm.2025.100199)
Supplement: 1 [file NIHMS2126146-supplement-1.docx]

**SUPPLEMENTAL DATA**

| Analysis | Comparison | Statistic | P-Value |
| --- | --- | --- | --- |
| Clinical Characteristics | Caffeine vs Non-Caffeine Users |  |  |
| - Inpatient at consult | 28.0% vs 51.1% | χ² = 7.19 | 0.007** |
| - Inpatient during treatment | 18.9% vs 48.9% | χ² = 14.4 | <0.001*** |
| - Past ECT/Ketamine | 22.0% vs 34.0% | χ² = 2.08 | 0.149 |
| - Failed antidepressant trials | 5.85 ± 3.25 vs 5.77 ± 3.06 | W = 3243.5 | 0.64 |
| - Baseline QIDS-SR16 | 16.08 ± 4.12 vs 15.64 ± 4.38 | W = 2912 | 0.533 |
| - Baseline PHQ-9 | 15.74 ± 5.50 vs 16.13 ± 4.75 | W = 3222.5 | 0.693 |
| - Baseline GAD-7 | 11.79 ± 5.58 vs 11.62 ± 5.37 | W = 3041.5 | 0.904 |
| Inpatient vs Outpatient  (Non-Caffeine, age/sex matched) | Inpatient (n = 23) vs Outpatient (n = 23) |  |  |
| - QIDS percent change | Mean % change | W = 239.5 | 0.59 |
| - PHQ-9 percent change | Mean % change | W = 205.5 | 0.199 |
| - QIDS response rate | Response rate | χ² = 1.49 | 0.222 |
| - PHQ-9 response rate | Response rate | χ² = 1.44 | 0.231 |
| - QIDS remission rate | Remission rate | χ² = 0.00 | 1 |
| - PHQ-9 remission rate | Remission rate | χ² = 1.58 | 0.208 |
| Inpatient vs Outpatient  (Caffeine, age/sex matched) | Inpatient (n = 37) vs Outpatient (n = 37) |  |  |
| - QIDS percent change | Mean % change | W = 633.5 | 0.585 |
| - PHQ-9 percent change | Mean % change | W = 617.5 | 0.472 |
| - QIDS response rate | Response rate | χ² = 0.97 | 0.326 |
| - PHQ-9 response rate | Response rate | χ² = 0.06 | 0.813 |
| - QIDS remission rate | Remission rate | χ² = 0.37 | 0.541 |
| - PHQ-9 remission rate | Remission rate | χ² = 0.00 | 1 |
| Caffeine vs Non-Caffeine  (Outpatients only) | Caffeine (n = 95) vs  Non-Caffeine (n = 23) |  |  |
| - QIDS percent change | Mean % change | W = 1065.5 | 0.857 |
| - PHQ-9 percent change | Mean % change | W = 1021.5 | 0.632 |
| - QIDS response rate | Response rate | χ² = 0.41 | 0.524 |
| - PHQ-9 response rate | Response rate | χ² = 0.49 | 0.485 |
| Caffeine vs Non-Caffeine  (Inpatients only) | Caffeine (n = 37) vs  Non-Caffeine (n = 24) |  |  |
| - QIDS percent change | Mean % change | W = 475.5 | 0.647 |
| - PHQ-9 percent change | Mean % change | W = 507.5 | 0.352 |
| - QIDS response rate | Response rate | χ² = 0.02 | 0.886 |
| - PHQ-9 response rate | Response rate | χ² = 0.07 | 0.799 |

**Supplemental Table 1.** *Statistical Analyses Examining Inpatient Status as a Confounder*
